# Characteristics of Lower Limb Muscle Activity During Walking and Stair Climbing With Full Weight Bearing in Postoperative Ankle Fracture Patients

**DOI:** 10.7759/cureus.87620

**Published:** 2025-07-09

**Authors:** Masanobu Yokochi, Masatoshi Nakamura, Ayaka Iwata, Ryota Kaneko, Naoyuki Oi, Kazuo Ouchi, Tetsuo Hayashi

**Affiliations:** 1 Rehabilitation, Takeda General Hospital, Aizuwakamatsu, JPN; 2 Rehabilitation Medicine, Fukushima Medical University School of Medicine, Fukushima, JPN; 3 Physical Therapy, Faculty of Rehabilitation Sciences, Nishikyushu University, Saga, JPN; 4 Community Health Care, Nagano University of Health and Medicine, Nagano, JPN

**Keywords:** ankle fracture, lower limb muscle activity, stairs, surface electromyography, walking

## Abstract

Background

Ankle fractures are among the most common lower limb fractures. Following surgery, the ankle is immobilized in a splint or cast for a specific period. Even after full weight bearing is allowed, daily activities, such as walking and stair climbing, may be affected by residual pain, limited joint range of motion (ROM), and muscle weakness. The present study aimed to investigate lower limb muscle activity during walking and stair climbing in patients capable of unassisted ambulation after ankle fracture surgery.

Methods

The study included 18 patients who underwent open osteosynthesis for unilateral ankle fractures at our hospital, and 18 healthy volunteers with no history of lower limb fractures as a control group. The mean age of the postoperative ankle fracture patients was 59.0 ± 12.4 years, compared to 62.7 ± 12.0 years for the healthy volunteers. The mean time between surgery and this measurement for ankle fracture patients was 72.7 ± 27.1 days. Ankle ROM, 10-meter walking speed, and lower limb muscle activity via surface electromyography were measured during walking and stair ascent/descent.

Results

Ankle ROM was significantly lower in the patient group than in the control group for both active and passive movements, as well as walking speed. Lower limb muscle activity during walking was higher in the patient group than in the control group for all muscles examined. Lower limb muscle activity during stair ascent was higher in the patient group. On the other hand, during stair descent, no significant differences were found between the two groups regarding vastus medialis (p = 0.195) and soleus (p = 0.121) muscle activity; however, the other muscles showed significantly higher activity in the patient group.

Conclusion

In the present study, ankle ROM, walking speed, and lower limb muscle activity during walking and stair climbing were compared between the patients capable of independent walking after ankle fracture surgery and healthy controls. Ankle ROM and walking speed were significantly lower in the patient group; however, lower limb muscle activity during both walking and stair climbing was higher in the patient group compared to the control group, suggesting decreased muscle strength in the patients and highlighting the need for appropriate training. Although rehabilitation was conducted, some muscles exhibited higher activity levels than others, indicating the need for exercises tailored to each muscle.

## Introduction

Ankle fractures are among the most common fractures of the lower limb [1]; their treatment is based on radiological findings, individual patient characteristics, and clinical symptoms, and includes a variety of conservative or surgical approaches. Following surgery, the ankle is immobilized in a splint or cast for some time, after which full weight bearing is introduced. Even after full weight bearing is allowed, patients may experience difficulties in activities of daily living due to pain, limited joint range of motion, and claudication. A previous study conducted a questionnaire survey on 50 postoperative ankle fracture patients aged ≥ 65 years, and reported that 60% of the patients had persistent pain, swelling, and difficulty climbing stairs 12 months postoperatively [2].

Various walking characteristics in patients following ankle fracture surgery have been reported in previous studies. A systematic review by Mirando et al. reported a decrease in stride length, stance time, and walking speed compared to healthy controls [3]. In a study of patients at six to eight weeks after ankle fracture surgery, stride length as well as stance and swing phase durations were significantly shorter compared to those of healthy controls [4]. In addition, a similar study, focusing on a 12-month postoperative study period, reported that ankle fracture patients had lower plantar pressures and shorter contact times during walking compared to healthy controls [5]. Thus, although studies investigating plantar pressure and range of motion (ROM) during walking were found, few papers have investigated muscle activity while walking in patients after ankle fracture surgery. Zhu et al. investigated lower limb muscle activity while walking in patients with trimalleolar ankle fractures postoperatively, using surface electromyography (sEMG), and reported higher tibialis anterior (TA) and peroneus longus (PL) muscle activity compared to healthy controls [6]. In our study, we also investigated lower extremity muscle activity during full weight-bearing walking and stair climbing in patients after trimalleolar fractures surgery, and found no improvement in vastus medialis (VM) muscle activity during full weight bearing and one month later [7]. These results suggest that ankle fractures may affect muscle activity in patients while walking. In addition, previous studies have reported that immobility during cast immobilization in ankle fracture patients reduces the thigh muscle cross-sectional area [8]. Therefore, muscle activity during walking may be altered in the thigh muscles as well as the lower leg muscles in patients with ankle fractures. Furthermore, as mentioned above, altered muscle activity may also occur during stair climbing in patients with ankle fractures. Elucidation of these changes in muscle activity is expected to contribute to the selection of rehabilitation interventions for ankle fracture patients in the future. Therefore, the present study aimed to investigate lower limb muscle activity during walking and stair climbing in patients capable of independent ambulation after ankle fracture surgery.

## Materials and methods

Participants

The study population comprised 18 patients who had visited our hospital between January 2022 and February 2023 and had undergone open osteosynthesis for a unilateral ankle fracture. None of the patients experienced surgical complications. The fracture types in the 18 patients were: lateral malleolus fracture in one, bimalleolar fracture in three, and trimalleolar fracture in 14. All patients were independently mobile before their injury. The mean age of the postoperative ankle fracture patients was 59.0 ± 12.4 years, compared to 62.7 ± 12.0 years for the healthy volunteers. The mean between surgery and this measurement for ankle fracture patients was 72.7 ± 27.1 days. The mean score on the Japanese version of the Lower Extremity Functional Scale [9] was 47.8 ± 9.0. The exclusion criteria were severe motor paralysis due to stroke or other causes, and lack of consent for participation in the study. After surgery, all patients were immobilized with a splint for a mean duration of 19.9 ± 7.6 days. Rehabilitation began on the day after surgery under the direction of the attending doctor, including stretching, muscle strengthening, and non-weight-bearing exercises with the use of crutches. The splint was then removed according to the doctor's instructions, and ankle ROM exercises were started on the injured side. Partial or full weight-bearing walking was allowed depending on X-ray findings, and weight-bearing exercises were performed in phases. The mean duration between surgery and achievement of independent ambulation was 72.7 ± 27.1 days. The mean duration from full weight bearing to independent ambulation was 25.5 ± 17.5 days. As a comparison group, 18 healthy volunteers with no lower limb fracture history were recruited. The recruitment criteria for healthy volunteers were age ≥ 45 years and no history of ankle injury or fracture. The application method was to post a recruitment poster at the affiliated facility. The dominant leg, defined as the leg naturally favored when kicking a ball, was the right leg for all healthy volunteers included in the study [10].

All participants were informed in writing of the detailed procedure and signed an informed consent form before measurements were taken. The study was approved and conducted by the Ethical Review Committee of Fukushima Medical University (approval number: General 2021-201).

Measurement parameters

Ankle joint ROM, lower limb muscle activity during walking and stair climbing, and 10-m walking speed as an index of motor function were measured in both patient and control groups. In addition, pain intensity during ROM measurement and walking was assessed in the patient group. Measurements for the patients were conducted on the day they achieved the ability to walk independently

Ankle joint ROM measurement

Active and passive ankle joint ROM in the sagittal plane (dorsiflexion and plantar flexion) was measured using a manual goniometer with the patient in the supine position. ROM was assessed by measuring the maximum extension angle of the participant’s ankle, first created actively by the participant, then passively by the physiotherapist [11, 12]. Dorsiflexion was measured with the knee extended and with the ankle in the neutral position. All measurements were taken with the participant in the supine position.

10-m walking speed

Participants were instructed to walk 10 m at a comfortable pace, and the time taken to do so was measured with a stopwatch. A 3-m buffer was established at the start and end of each measurement. Measurements were taken twice, and the average was taken.

Pain intensity measurement

Ankle pain in the patients was assessed using a visual analog scale (VAS) during active/passive ROM measurement for dorsiflexion and plantar flexion, as well as during the 10-m walking test. Patients were asked to rate their pain by marking an X on a 100-mm VAS (0 mm for no pain, 100 mm for worst possible pain). The VAS has high test-retest reliability (intra-class correlation coefficient = 0.94) and has been shown to correlate with other tests of pain intensity [13].

Muscle activity measurements during a walk via sEMG

The participants were instructed to walk at a normal speed with their shoes on in the rehabilitation room. sEMG-connected footswitches (Marq-Medical, Denmark) were placed at the heel and great toe metatarsophalangeal joint on the affected side to measure the walking cycle. The footswitch becomes conductive by applying pressure to a sensitive, 1-cm diameter area and produces a square wave output. Data from 10 walking cycles were used for sEMG analysis during walking, as previously reported [14].

sEMG was measured by telemetry using MQ-Air2 (KISSEI COMTEC Co., Ltd., Nagano, Japan) and its recording software (Vital Recorder2; KISSEI COMTEC Co., Ltd., Nagano, Japan). The measurement was performed using the bipolar deviation method with an inter-electrode distance of 2 cm. sEMG waveforms were collected at a sampling frequency of 1 kHz, and analyzed using a multipurpose bioinformation analysis system (Bimutas Video; KISSEI COMTEC Co., Ltd., Nagano, Japan) followed by conversion to full-wave rectified waveforms. Amplified signals (raw sEMG) were processed using the root mean square method. Disposable circular monopolar Ag/AgCl electrodes (diameter 10 mm) were used according to the guidelines of the Surface Electromyography for the Non-Invasive Assessment of Muscles (SENIAM). The electrode distance was fixed at 2 cm [15]. Muscle activity measurement via sEMG was performed for rectus femoris (RF), vastus lateralis (VL), VM, TA, PL, soleus (Sol), and gastrocnemius lateral (GL) muscles. The participant’s skin was prepared for electrode placement. The muscle electrodes were placed according to the SENIAM guidelines [3] [16]. The RF electrode was placed at the distal half of the line connecting the anterior superior iliac spine and the upper edge of the patella; the VL electrode was placed at the distal third of the line connecting the anterior superior iliac spine and the lateral patella; the VM electrode was placed at the distal fifth of the line connecting the anterior superior iliac spine and the knee joint fissure. The TA electrode was placed on the proximal third of the line connecting the head of the fibula to the tip of the medial condyle; the PL electrode was placed on the proximal quarter of the line connecting the head of the fibula to the tip of the external capsule; the Sol electrode was placed at the distal third of the line connecting the medial femoral condyle to the medial capsule; and the GL electrode was placed at the proximal third of the line connecting the head of the fibula to the heel. Subsequently, the maximum voluntary isometric contractions (MVICs) of these muscles were measured for five sec, with a one minute rest period between each measurement, and were utilized for normalizing sEMG signals [17]. MVICs of the quadriceps were performed with the patient in a seated position. The participant maintained their lower leg in a slightly flexed position, with the leg bent from the knee, keeping the trunk in the midline position by placing their hands flat on the sitting surface on each side, while the examiner applied manual resistance to the distal upper surface of the lower leg. For TA, the participant sat with their lower limbs naturally hanging down, maintaining maximum ankle dorsiflexion, while the examiner applied manual resistance to the dorsal aspect of the foot. For PL, the participant sat with their lower limbs naturally hanging down, maintaining maximum ankle eversion, while the examiner applied manual resistance to the lateral aspect of the forefoot. Regarding Sol and GL MVICs, the participant stood without support and maintained their ankles in plantar flexion, while the examiner placed both hands on the participant's shoulders and applied downward resistance. Manual resistance during MVIC measurements was applied for three sec each time. All MVIC measurements were repeated twice, with a two minute rest period between each one. Finally, MVICs and EMG signals during walking were processed by filtering, full-wave rectification, and smoothing. The maximum value of sEMG signals was used for normalization during walking [18].

Muscle activity measurements during stair climbing

Lower limb muscle activity during stair climbing was measured on staircases of four 15-cm-high steps in the rehabilitation room. Participants were not allowed to use handrails, walking sticks, or any other kind of support during the measurement. First, the lower limb muscle activity of the ascending step was measured, followed by the lower limb muscle activity of the descending step. Data from three ascending steps and three descending steps, specifically from the second to fourth steps, inclusive, were used for analysis.

Statistical analysis

Analyses were performed using SPSS Software (version 28.0; IBM Corp., Armonk, NY, USA). The unpaired t-test was used to compare the patients and healthy controls. The effect size was calculated using Cohen’s d, whose values are quantified as follows: 0.2 small, 0.5 medium, and 0.8 large [19]. The effect size (Cohen’s d), calculated as the difference of the means of two groups divided by the weighted pooled standard deviations of these groups.

## Results

The demographic characteristics of the participants are shown in Table 1.

**Table 1:** Baseline characteristics of participants (mean ± SD) n: number of participants; BMI: body mass index. Results are expressed as mean (standard deviation) unless otherwise indicated. Statistical significance was set at p < 0.05.

	Postoperative ankle fracture patients (n = 18)	Healthy controls (n = 18)	test statistic	p value
Age (years)	59.0±12.4	62.7±12.0	1.689	0.367
Height (cm)	163.9±10.3	161.0±8.6	1.049	0.302
Weight (kg)	67.9±10.3	55.8±7.5	3.38	0.001
BMI (kg/㎡)	25.2 ± 4.2	21.7±2.5	2.998	0.005
Male/female	12/6	7/11	2.786	0.1

Table 2 shows measurement results of ankle ROM and walking speed in the patient and control groups.

**Table 2:** Ankle joint ROM and 10-m walking speed ROM: range of motion. Results are expressed as mean (standard deviation), unless otherwise indicated. Statistical significance was set at p < 0.05.

	Postoperative ankle fracture patients (n=18)	Healthy controls (n=18)	t value	p value	d value
Active ROM dorsifexion (degree)	5.6±4.1	16.7±3.7	-8.33	<0.01	2.777
Passive ROM dorsifexion (degree)	6.9±4.1	16.7±3.7	-7.206	<0.01	2.402
Active ROM plantar fexion (degree)	43.9±12.3	56.4±7.8	-3.539	0.001	1.18
Passive ROM plantar fexion (degree)	47.2±10.2	56.4±7.8	-2.952	0.006	0.984
10-m walking test (m/s)	0.8±0.2	1.3±0.2	-7.737	<0.01	2.579

Statistical analysis showed no significant differences in age or gender between the groups, although BMI was significantly higher in the patient group. Ankle ROM for both active and passive movements and walking speed were significantly lower in the patient group. Ankle pain on a 100-mm VAS during ROM measurement and walking was: 10.0 ± 9.6 mm for active dorsiflexion; 12.3 ± 10.4 mm for passive dorsiflexion; 5.0 ± 6.9 mm for both active and passive plantar flexion; and 13.4 ± 10.9 mm for walking. Lower limb muscle activity during walking and stair climbing is shown in Tables 3, 4, 5.

**Table 3:** Muscle activity during walking n: number of participants. Results are expressed as mean (standard deviation), unless otherwise indicated. Statistical significance was set at p < 0.05.

	Postoperative ankle fracture patients (n=18)	Healthy controls (n=18)	t value	p value	d value
Rectus femoris muscle	30.8±15.4	15.6±5.8	3.92	<0.01	1.307
Vastus lateralis muscle	31.2±12.7	21.1±3.7	3.13	0.005	1.043
Vastus medialis muscle	37.1±20.0	21.6±6.8	3.113	0.005	1.038
Tibial anterior muscle	27.2±14.6	14.4±6.2	3.34	0.003	1.113
Peroneal longus	62.2±23.2	32.9±6.3	5.041	<0.01	1.68
Soleus muscle	39.2±20.1	26.1±3.5	2.735	0.014	0.912
Gastrocnemius lateral	45.5±16.1	26.8±4.6	4.731	<0.01	1.577

**Table 4:** Lower limb muscle activity during stair ascent n: number of participants. Results are expressed as mean (standard deviation), unless otherwise indicated. Statistical significance was set at p < 0.05.

	Postoperative ankle fracture patients (n=18)	Healthy controls (n=18)	t value	p value	d value
Rectus femoris muscle	31.8±20.4	20.3±5.9	2.29	0.033	0.763
Vastus lateralis muscle	35.3±14.5	26.6±1.3	2.407	0.025	0.802
Vastus medialis muscle	38.7±23.8	26.2±7.7	2.114	0.047	0.705
Tibial anterior muscle	27.5±16.5	17.6±6.3	2.311	0.031	0.77
Peroneal longus muscle	64.6±25.9	39.2±8.5	3.957	<0.01	1.319
Soleus muscle	45.1±21.9	30.6±5.3	2.724	0.013	0.908
Gastrocnemius lateral muscle	47.7±20.1	31.8±5.7	3.223	0.004	1.074

**Table 5:** Lower limb muscle activity during stair descent n: number of participants. Results are expressed as mean (standard deviation), unless otherwise indicated. Statistical significance was set at p < 0.05.

	Postoperative ankle fracture patients (n=18)	Healthy controls (n=18)	t value	p value	d value
Rectus femoris muscle	29.4±12.8	18.8±7.1	2.956	0.006	0.985
Vastus lateralis muscle	30.4±11.1	23.7±5.4	2.309	0.03	0.77
Vastus medialis muscle	28.9±11.8	24.3±9.0	1.323	0.195	0.441
Tibial anterior muscle	21.6±10.0	13.5±5.9	2.95	0.006	0.983
Peroneal longus muscle	65.5±22.9	36.4±9.7	4.96	<0.01	1.653
Soleus muscle	38.8±23.3	29.4±5.9	1.624	0.121	0.541
Gastrocnemius lateral muscle	47.5±18.6	27.8±6.5	4.233	<0.01	1.411

Statistical analysis revealed that lower limb muscle activity during both walking and stair ascent was significantly higher in the patient group for all muscles. On the other hand, there were no differences between the two groups regarding VM (p = 0.195) and Sol (p = 0.121) during stair descent, although the remaining muscles were significantly more active in the patient group.

## Discussion

The present study compared lower limb muscle activity, walking speed, and ankle ROM during full-load walking and stair climbing between postoperative ankle fracture patients and healthy controls. The results showed that 10-m walking speed and ankle ROM were significantly lower in the patient group than in the control group. Lower limb muscle activity during walking was higher in the patient group for all muscles measured. Lower limb muscle activity during stair ascent and descent was also higher in the patient group for all muscles except VM and Sol during descent. To the best of our knowledge, this is the first study to investigate lower limb muscle activity and walking speed in patients who reached full weight bearing after ankle fracture surgery.

Ankle dorsiflexion and plantar flexion ROMs were significantly lower in the patient group for both active and passive movements. Ankle ROM has also been investigated in previous studies, with Zhu et al. reporting a dorsiflexion ROM of 7.08 ± 3.91° and a plantar flexion ROM of 33.33 ± 8.07° at 4.5 months postoperatively [6]. In another study, ankle dorsiflexion ROM was 22.8 ± 7.7° at six months postoperatively [20]. The dorsiflexion ROM of the postoperative ankle fracture patients in the present study was 5.6 ± 4.1° for active movement and 6.9 ± 4.1° for passive movement, which were lower than those reported in previous studies. This may be due to the shorter time between surgery and ankle ROM measurements in the present study.

Walking speed was also lower in the patient group compared to the control group in the present study. Hsu et al. investigated 5-m walking speed in patients who had undergone ankle fracture surgery four months previously, and reported that it was lower compared to that of healthy controls. [21]. The walking speed of ankle fracture patients in their study was 0.74 m/s, which was similar to that of the patient group in the present study, at 0.8 m/s. Although the postoperative periods in Hsu et al.’s study and the present study differed, the measurement timings were consistent; i.e., when the patients could walk 10 m without walking aids, which may have contributed to similar results.

In the present study, lower limb muscle activity during walking was significantly higher in the patient group than in the control group. Yamauchi et al. reported that the thigh muscle cross-sectional area was approximately 28 days after cast immobilization for ankle fracture injury than immediately before injury [8]. Psatha et al. also investigated lower leg muscle cross-sectional areas for TA, GL, gastrocnemius medialis (GM), and soleus muscles in patients after six-week cast immobilization for ankle fracture, and reported that it was decreased in all muscles [22]. In the present study, splint immobilization was performed for approximately three weeks. This may reflect a decrease in lower limb muscle cross-sectional area, possibly resulting in a decrease in muscle strength and an increase in lower limb muscle activity during walking. Zhu et al.’s study investigated the lower limb muscle activity of TA, PL, GL, GM, and Sol during walking in ankle fracture patients and healthy controls, and reported that TA and PL muscles showed higher activity in ankle fracture patients [6]. In the present study, the mean age of the patients was 59.0 ± 12.4 years, which is younger than that of Zhu et al.’s study (42.20 ± 10.20 years). In addition, while the mean postoperative period until measurement was 4.5 months in the previous study, it was 2.5 months in the present study, which could have resulted in the patients being more susceptible to the impact of surgical invasion, resulting in higher lower limb muscle activity.

Lower limb muscle activity during stair ascent and descent was significantly higher in the patient group, except for VM and Sol during descent. Shaffer et al. conducted a study comparing the peak torque of the ankle plantar flexors during stair ascent and descent between post-ankle surgery patients and healthy controls, and reported that it was lower in post-ankle surgery patients [23]. As mentioned above, we hypothesize that higher muscle activity of the lower limb in the patient group may have been affected by muscle atrophy and weakness. On the other hand, VM and Sol showed no difference in muscle activity between the two groups. A previous study investigating muscle activity during stair ascent and descent in healthy individuals in their 20s and those in their 70s healthy individuals reported no differences regarding muscle activity in Sol [24], and that differences in muscle activity between ankle fracture patients and healthy subjects are unlikely.

A limitation of the present study is that the classification and severity of ankle fractures were not taken into account. Previous studies have reported that patients with trimalleolar fractures have significantly lower ROM between the hindfoot and tibia in the sagittal plane (flexion/extension) during the push-off phase than patients with Unimalleolar fractures. In addition, patient-reported outcome measures (PROM) also showed that patients with trimalleolar fractures had the worst outcomes compared to patients with unimalleolar or bimalleolar fractures [10]. Therefore, future studies should be conducted according to fracture severity．

## Conclusions

In the present study, ankle ROM, walking speed, and lower limb muscle activity during walking and stair climbing were compared between patients who achieved the ability to walk unassisted after ankle fracture surgery and healthy controls. The results showed that ankle ROM and walking speed were significantly lower in the patient group. For all lower limb muscle activities, except VM and Sol muscle activity during stair descent, the patient group showed significantly higher muscle activity than the control group. The results of the present study characterize lower extremity muscle activity during walking and stair climbing in postoperative ankle fracture patients. Rehabilitation should take into account the lower extremity muscle activity reduction and ROM limitation in such patients.
